# Grouping of multicopper oxidases in *Lentinula edodes* by sequence similarities and expression patterns

**DOI:** 10.1186/s13568-015-0151-2

**Published:** 2015-09-17

**Authors:** Yuichi Sakamoto, Keiko Nakade, Kentaro Yoshida, Satoshi Natsume, Kazuhiro Miyazaki, Shiho Sato, Arend F. van Peer, Naotake Konno

**Affiliations:** Iwate Biotechnology Research Center, 22-174-4 Narita, Kitakami-shi, Iwate 024-0003 Japan; TSUMURA and Co., 3586 Yoshiwara, Ami-machi, Inashiki-gun, Ibaraki 300-1192 Japan; Graduate School of Agricultural Science, Kobe University, 1-1 Rokkodai, Nada-ku, Kobe, 657-8501 Japan; Kyushu Research Center Forestry and Forest Products Research Institute, 4-11-16 Kurokami, Kumamoto, Kumamoto 860-0862 Japan; College of Life Sciences, Fujian Agriculture and Forestry University, Fuzhou, 350002 People’s Republic of China; Faculty of Agriculture, Utsunomiya University, 350 Mine-machi, Utsunomiya, Tochigi 321-8505 Japan

**Keywords:** Fruiting body, Laccase, *Lentinula edodes*, Lignin degradation, Multicopper oxidase

## Abstract

The edible white rot fungus *Lentinula edodes* possesses a variety of lignin degrading enzymes such as manganese peroxidases and laccases. Laccases belong to the multicopper oxidases, which have a wide range of catalytic activities including polyphenol degradation and synthesis, lignin degradation, and melanin formation. The exact number of laccases in *L. edodes* is unknown, as are their complete properties and biological functions. We analyzed the draft genome sequence of *L. edodes* D703PP-9 and identified 13 multicopper oxidase-encoding genes; 11 laccases in sensu stricto, of which three are new, and two ferroxidases. *lcc8*, a laccase previously reported in *L. edodes*, was not identified in D703PP-9 genome. Phylogenetic analysis showed that the 13 multicopper oxidases can be classified into laccase sensu stricto subfamily 1, laccase sensu stricto subfamily 2 and ferroxidases. From sequence similarities and expression patterns, laccase sensu stricto subfamily 1 can be divided into two subgroups. Laccase sensu stricto subfamily 1 group A members are mainly secreted from mycelia, while laccase sensu stricto subfamily 1 group B members are expressed mainly in fruiting bodies during growth or after harvesting but are lowly expressed in mycelia. Laccase sensu stricto subfamily 2 members are mainly expressed in mycelia, and two ferroxidases are mainly expressed in the fruiting body during growth or after harvesting, and are expressed at very low levels in mycelium. Our data suggests that *L. edodes* laccases in same group share expression patterns and would have common biological functions.

**Electronic supplementary material:**

The online version of this article (doi:10.1186/s13568-015-0151-2) contains supplementary material, which is available to authorized users.

## Introduction

White-rot fungi produce a variety of extracellular lignin degrading enzymes, including lignin peroxidases, manganese peroxidases, and laccases. These enzymes have been receiving widespread attention because of their ability to degrade environmentally persistent xenobiotics as well as endocrine-disrupting chemicals like pentachlorophenol, polychlorinated biphenyls, and dioxins (Jeon et al. 2008; Fujihiro et al. 2009; Ullah et al. 2000).

Laccases (EC 1.10.3.2) belong to a group of polyphenol oxidases that contain copper atoms in their catalytic center and are therefore typically referred to as multicopper oxidases. They catalyze single-electron oxidation of phenolic substrates or aromatic amines, resulting in numerous products (Leonowicz et al. 2001). While plant laccases are mainly involved in formation of lignin polymers in a radical-based mechanism (Hoopes and Dean 2004; Ranocha et al. 2002; Sterjiades et al. 1992), fungal laccases have been reported to play roles in lignin degradation (Baldrian 2006; Thurston 1994), cell wall formation (Nakade et al. 2011), pathogenicity (Nosanchuk and Casadevall 2003) and fruiting body coloration (Nagai et al. 2003). The number of reports on the purification and characterization of laccases from white-rot fungi is becoming extensive (Baldrian 2006; Kumar et al. 2003); and includes reports on *Trametes versicolor* (Galhaup et al. 2002; Jönsson et al. 1997), *Pleurotus ostreatus* (Palmieri et al. 2000), and *Lentinula edodes* (Nagai et al. 2002, 2003). Sequence comparison and transcription analysis has been carried out in several basidiomycetous fungi as well, such as *Coprinopsis cinerea* (Kilaru et al. 2006), *P. ostreatus* (Castanera et al. 2012; Pezzella et al. 2013), *Flammulina velutipes* (Wang et al. 2015) and *Laccaria bicolor* (Courty et al. 2009) and *Schizophyllum commune* (Madhavan et al. 2014). Recently, it was revealed that most basidiomyceteous fungi have more than 10 genes that encode different laccases in their genome (Floudas et al. 2012). Most laccases have been purified as secreted enzymes from fungal mycelia or as expressed recombinant enzymes, though some have been extracted from fruiting bodies (Lettera et al. 2010; Nagai et al. 2003). While these reports have generated detailed insights on sequence, structure, function and expression of specific laccases, an overall understanding of the relationship among biological spectrum, sequence similarity and expression pattern is still limited.

*Lentinula edodes* (*Marasmiaceae*), or shiitake as the mushroom is more popularly known, is one of the most important cultivated edible mushrooms as well as a white-rot fungus that degrades lignin in wood. Two laccases, Lcc1 (Nagai et al. 2002; Zhao and Kwan 1999) and Lcc4 (previously designated Lcc2; Nagai et al. 2003), have been purified from *L. edodes* and the corresponding genes have been cloned (Sakamoto et al. 2008, 2009). Nagai et al. (2009) purified another *L. edodes* laccase, Lcc6. Additional laccase encoding genes (*lcc7*–*11*) were reported in *L. edodes* strain L45A, and Lcc1, Lcc4, Lcc5, and Lcc7 were expressed in *Pichia pastoris* for characterization (Wong et al. 2013). More recently, genome sequence data in several species in the *Marasmiaceae*, to which *L. edodes* belongs, have become available in public databases for *Omphalotus olearius*, *Dendrothele bispora*, *Moniliophthora perniciosa*, and *Gymnopus luxurians*, allowing comparison of laccases among closely related species. In this report, we analyzed the draft genome sequence of *L. edodes* strain D703PP-9 and identified a total of 13 multicopper oxidases, including 3 novel laccase encoding genes (*lcc12*–*14*), and 2 genes (*lcc10* and *lcc11* in Wong et al. 2013) that were later excluded from laccase sensu stricto subfamilies. Subsequent analysis of transcription patterns and phylogenetic relationships revealed that multicopper oxidases in *L. edodes* can be classified into 7 members of laccase sensu stricto subfamily 1 (and can be divided into two subgroups), 4 members of laccase sensu stricto subfamily 2, and two ferroxidases (*lcc10* and *lcc11*). The relationship between their sequence similarities and biological functions is discussed.

## Materials and methods

### Strains and culture conditions

*Lentinula edodes* monokaryotic strain D703PP-9 (Miyazaki et al. 2008; ICMP No. 20921) was used for genome sequence analysis. *L. edodes* D703PP-9 and monokaryotic strain G408PP-4 (Miyazaki et al. 2008; NBRC No. 111202) were used for linkage mapping. A dikaryotic, commercially cultivated strain, *L. edodes* H600 (obtained from Hokken Co., Ltd, Tochigi, Japan: Sakamoto et al. 2008, 2009), was used for isolating RNA of multicopper oxidase-encoding genes. Mycelial cultures on sawdust media were prepared as described in Sakamoto et al. (2005). Growing fruiting bodies were prepared as described in Sakamoto et al. (2005), and for post-harvest analysis, harvested mature fruiting bodies were immediately transferred to a desiccator at 25 °C (Sakamoto et al. 2005) and sampled daily from day 0 (fresh) to day 4. Upon sampling, mushrooms were separated into pileus, gill and stipe, and immediately frozen in liquid nitrogen.

### RNA and cDNA preparation

For RNA extraction, mycelia were cultured in MYPG liquid medium at 25 °C while shaking as described previously (Sakamoto et al. 2005). To extract RNA from mycelia grown on sawdust medium, a filter membrane (Isopore^TM^ Membrane Filter; Millipore, MA, USA) was placed on the sawdust and covered with 1.5 % agar. Mycelia from sawdust cultures were harvested 2 weeks after inoculation from the surface of the filter membrane. To extract RNA from fruiting bodies, primordia and fruiting bodies were prepared as described previously (Hirano et al. 1999; Nagai et al. 2003). cDNA was synthesized with a QuantiTect Reverse Transcription Kit (QIAGEN GmbH, Germany) following the manufacturer’s instructions.

### Genome sequencing of *L. edodes* D703PP-9

Genomic DNA was extracted from 2-week-old liquid cultures after crushing the mycelia in liquid nitrogen and using a MasterPure Yeast DNA Extraction Kit (Epicentre Biotechnologies, WI, USA) following the manufacturer’s instructions. Libraries for genome sequencing were prepared using a TruSeq DNA Sample Prep Kit v2 (Illumina, CA, USA) and 76 bp paired-end sequencing was performed with an Illumina Genome Analyzer IIx system. De novo sequences were assembled using Velvet assembler version 0.7.34 (2) (http://www.ebi.ac.uk/~zerbino/velvet/) by varying several parameters. We chose a set of contigs created under the conditions generating the longest N50 for further analyses. The blastx algorithm (ftp://ncbi.nlm.nih.gov/blast/executables/blast+/LATEST/) was used for identification of laccases. To predict transcripts for laccase encoding genes, the WISE2 algorithm was used (http://www.ebi.ac.uk/Tools/psa/genewise/).

### Cloning and sequencing of multicopper oxidase-encoding genes

Sequence data of *lcc1* through *lcc6* has been deposited in DDBJ (accession numbers Lcc1: AB822542; Lcc2: AB822543; Lcc3: AB822544; Lcc4: AB822545; Lcc5: AB822546; Lcc6: AB822547), and *lcc1* and *lcc4* have been previously reported (Sakamoto et al. 2008, 2009). For cloning of *lcc7* through *lcc14* (Lcc7: AB822548; Lcc9: AB822552; Lcc12: AB822549; Lcc13: AB822550; Lcc14: AB822551), we amplified the genes from cDNA synthesized from *L. edodes* H600 RNA extracted from mycelia or fruiting bodies. Open reading frames of *lcc7*, *lcc9,* and *lcc12* through *lcc14* were amplified using primer sets listed in Additional file 1: Table S1 and Ex Taq polymerase (TaKaRa, Bio. Inc., Kyoto, Japan), then sequenced using an AB3130XI sequencer (Applied Biosystems, CA, USA). Signal peptides of each laccase were predicted using SignalP (http://www.cbs.dtu.dk/services/SignalP/) algorithm.

### Phylogenetic analysis

Multicopper oxidase-encoding genes were analyzed phylogenetically by alignment of the respective amino acid sequences (data set Additional file 1: Table S2) using ClustalW software (http://www.ddbj.nig.ac.jp/search/clustalw-j.html). The phylogram was constructed using the neighbor-joining method, and trees were drawn using FigTree (http://tree.bio.ed.ac.uk/software/figtree/). Bootstrapping was carried out with 1000 replications.

### Protein detection and purification

Anti-sera for Lcc1 and Lcc4 were respectively described in Sakamoto et al. (2008) and Yano et al. (2009). The peptides used in the immunization (custom service of TakaRa Bio. Inc., Kyoto, Japan) were designed using the Epitope Adviser 2.1 program (FQS, Fukuoka, Japan) to identify putative Lcc2 epitopes (NVQQGKRYRFRMISIACDA, TGGLNSGILRYQGAPDADP and RSADNTTYNYKNPVRRD), Lcc3 epitopes (GAPEEEPQTSQPLSSN, TSDSSEYNFKNPVRRD, EDTRDTKKDDMIPAD), and Lcc5 epitopes (CSEPGTPEVTSVLALNE, CQLVPLENPGAPGEPE, and EDVADWNTTQTPSTAWDDC). Western blot analysis was carried out following the procedure described in Sakamoto et al. (2008). Purification steps for Lcc2, Lcc4, Lcc5 and Lcc13 are summarized in Additional file 1: Table S3, and amino acid sequences were determined using an ABI Procise 491HT Protein Sequencing System (Applied Biosystems, CA, USA). Laccase activity was measured following the methods described in Nagai et al. (2002).

### Linkage mapping of multicopper oxidase-encoding genes

For single-strand conformation polymorphism analysis, biotin-labeled PCR products were diluted 50- to 100-fold in 1× TBE buffer (89 mM Tris–HCl, pH 8.0, 89 mM boric acid, 2 mM EDTA), 6 % (w/v) sucrose, and 0.33 % tartrazine. DNA in the diluted solution was denatured at 96 °C for 5 min, cooled on ice and loaded on a 15 × 40 cm vertical 5 % HydroLink Long Ranger polyacrylamide gel (AT Biochem Malvern, PA, USA) in 1× TBE buffer, then electrophoresed at 14 °C for 90 min at 30 W. DNA was transferred to MSI nylon membranes (MSI, MA, USA) and visualized using a Phototope-Star Detection Kit (New England Biolabs, MA, USA).

Allele-specific PCR was performed in 20 mM Tris–HCl (pH 8.5), 50 mM KCl, 2.5 mM MgCl_2_, 0.16 mM each dNTP, 0.08 μM each primer, 5 ng genomic DNA, and 0.25 units Platinum Taq DNA polymerase (Invitrogen, CA, USA) in a total volume of 12.5 μL overlaid with mineral oil. The thermal cycling program (performed on a PE480 instrument, PerkinElmer **(**PerkinElmer, MA, USA) consisted of denaturation for 1 min at 95 °C, 35 cycles of 95 °C for 30 s, 55 °C for 90°s, 72 °C for 30 s, and a 10 min final incubation at 72 °C with subsequent cooling to 4 °C. The sequences of the primers used are shown in Additional file 1: Table S4.

Only the data for segregation in a 2:2 ratio in each tetrad was used for linkage analysis. The linkage of the markers was tested based on the LOD score (the threshold of the LOD score was 3.0, and the maximum distance was 25 cM). The MapMaker version 3.0 computer program was used for linkage analysis. In this program, an efficient algorithm that allowed a simultaneous multipoint analysis of any number of loci (Lander et al. 1987) was used. The loci were grouped using the “GROUP” (two-point analysis) and “COMPARE” commands. The Kosambi mapping function (Kosambi 1943) was applied to determine the distance between two loci.

### Analysis of expression of multicopper oxidase-encoding genes by real-time PCR

The expression of *lcc1* in mycelia, young fruiting bodies during development, and fruiting bodies after harvest was analyzed by real-time PCR. Total RNA was isolated using the MasterPure Yeast RNA Extraction Kit (Epicentre Biotechnologies, WI, USA) and reverse transcribed using a QuantiTect kit (QIAGEN GmbH, Germany) according to the manufacturer’s instructions. Real-time PCR was performed using SYBR Premix Ex Taq reaction solution (TaKaRa, Bio. Inc., Kyoto, Japan) and a StepOne Plus real-time PCR system (Applied Biosystems, CA, USA). To analyze the level of transcription of *lcc1* through *14* (except for *lcc8*: Wong et al. 2013), we used the primer sets listed in Additional file 1: Table S5. We also analyzed the expression of *gpd* (Hirano et al. 1999) as an internal control using the primers gpd-rtU and gpd-rtL (Sakamoto et al. 2008). To standardize the results, mRNA levels of multicopper oxidase-encoding genes were determined based on the ratio between the transcript levels of these multicopper oxidase-encoding genes and *gpd*. The expression patterns were analyzed by ΔΔCT method (Livak and Schmittgen 2001) with three replicates, and the expression level of mycelia cultivated in liquid medium was used as a calibrator.

## Results

### Prediction of multicopper oxidases from the draft sequence of *L. edodes*

**Fig. 1:**
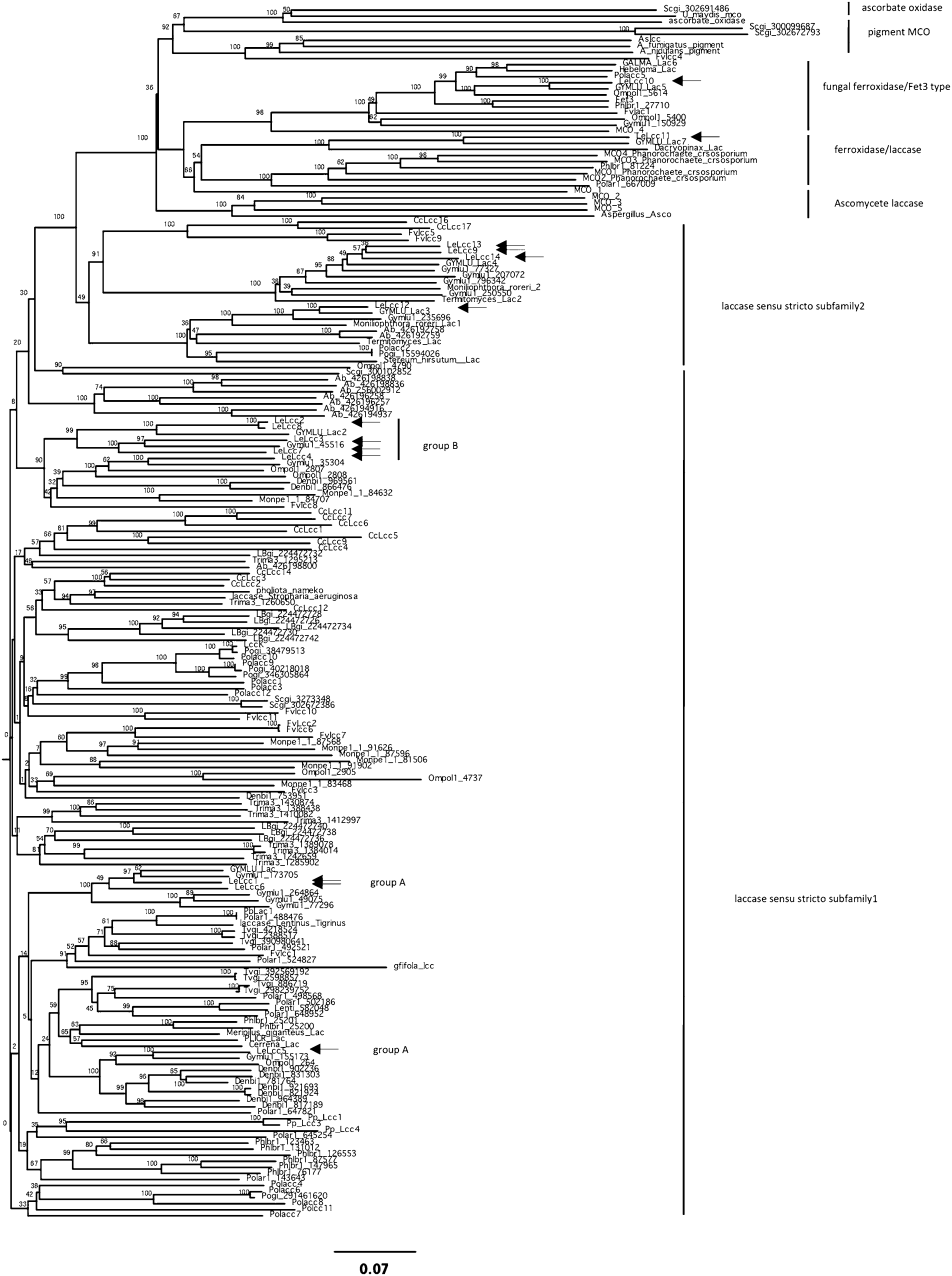
Phylogenetic analysis of laccases in *L. edodes*. Accession numbers of each multicopper oxidase gene are summarized in Additional file 1: Table S2. The phylogram was constructed using the neighbor-joining method. The *scale bar* indicates a distance of 0.07, and the *numbers on branches* indicate percentage bootstrap support values (based on 1000 replications). Multicopper oxidases in *L. edodes* are indicated with *arrows*

In order to identify multicopper oxidases including laccases in *L. edodes* D703PP-9 (Miyazaki et al. 2008), we obtained a draft genome sequence based on de novo assembly of short read sequences from an Illumina genome analyzer (GAIIx). The total length of the resulting contigs was approximately 35.5 Mbp (Table 1), which is roughly similar to the genome size of the basidiomycetous mushroom fungi *C. cinerea* (37.5 Mbp, Stajich et al. 2010)*, S. commune* (38.5 Mbp, Ohm et al. 2010), and *Agaricus bisporus* (30.0 Mbp, Morin et al. 2012), yet smaller than that of *L. bicolor* (60.7 Mbp, Martin et al. 2008). The quality of the draft genome (assembled contigs) was assessed through blastx searches against known *L. edodes* genes available in protein databases (336 proteins). Of these proteins, 99.5 % (321/336) were represented in the draft genome, indicating sufficient quality for gene identification in *L. edodes*. Next, we predicted multicopper oxidase-encoding genes based on sequence similarities, and full-length multicopper oxidase-encoding genes were curated using the GeneWise algorithm. From the predicted multicopper oxidase-encoding genes of D703PP-9, we found *lcc1* through *11* except for *lcc8* (Additional file 1: Table S6, Wong et al. 2013), and 3 new laccase encoding genes (*lcc12* through *lcc14*). These genes (*lcc1* through *14*, except for *lcc8*) were deposited in DDBJ (accession numbers Lcc1: AB822542; Lcc2: AB822543; Lcc3: AB822544; Lcc4: AB822545; Lcc5:AB822546; Lcc6:AB822547; Lcc7:AB822548; Lcc9: AB822552; Lcc10: LC018712; Lcc11: LC018713; Lcc12: AB822549; Lcc13: AB822550; Lcc14: AB822551). To validate the gene models for the newly identified laccases (*lcc12* through *lcc14*), we cloned the respective encoding genes (accession numbers: Lcc7: AB821483; Lcc9: AB821487; Lcc12: AB821484; Lcc13: AB821485; Lcc14: AB821486) newly from *L. edodes* strain H600 and sequenced the cDNA of each gene to confirm its open reading frame, the number of introns and the promoter region (Additional file 1: Table S7). Sequence similarities among the putative amino acid sequences of the cDNAs of these multicopper oxidase-encoding genes are summarized in Table 2, which shows that several multicopper oxidases shared higher sequence similarity and can be clustered. Lcc1 and Lcc6 had the highest similarity (81.4 %), and Lcc5 had higher similarity to Lcc1 (67.1 %) and *lcc6* (65.6 %) compared with other multicopper oxidases. Lcc2, Lcc3, Lcc4 and Lcc7 shared higher similarity with each other (62–69 %). Lcc9, Lcc13 and Lcc14 also shared the highest similarity with each other (84.5–87 %). On the other hand, Lcc10, Lcc11 and Lcc12 had lower sequence similarity to all other multicopper oxidases in *L. edodes* (25–30 % in Lcc10 and Lcc11, and 27–47 % in Lcc12; Table 2). Phylogenetic analysis (Fig. 1) revealed that multicopper oxidases in *L. edodes* could be categorized into laccase sensu stricto subfamily 1 (Lcc1, Lcc5, Lcc6, Lcc2, Lcc3, Lcc4 and Lcc7), laccase sensu stricto subfamily 2 (Lcc9, Lcc12 Lcc13 and Lcc14) and ferroxidases (Lcc10 and Lcc11) following the previous classification of multicopper oxidases (Kües and Rühl 2011; Hoegger et al. 2006).

**Table 1:** Summary of draft genome sequence of *L. edodes* strain D703P-9

Analysis	Results
No of contigs	35,534
Sum of bps (bp)	35,696,002
Max length (bp)	219,501
Min length (bp)	69
n50 (bp)	23,581
Putative ORF	8271

**Table 2:** Similarities among putative amino acid sequences of multi copper oxidases in *Lentinula edodes*

	Lcc1 (%)	Lcc5 (%)	Lcc6 (%)	Lcc2 (%)	Lcc3 (%)	Lcc4 (%)	Lcc7 (%)	Lcc12 (%)	Lcc13 (%)	Lcc14 (%)	Lcc9 (%)	Lcc10 (%)	Lcc11 (%)
Lcc1													
Lcc5	67.10												
Lcc6	81.40	65.60											
Lcc2	61.60	58.80	60.20										
Lcc3	59.10	54.70	55.90	66.70									
Lcc4	61.30	56.10	59.90	62.50	63.40								
Lcc7	57.90	55.10	56.20	66.30	69.20	63.10							
Lcc12	49.90	49.90	48.40	48.70	48.10	50.20	47.70						
Lcc13	42.10	41.70	40.30	42.40	43.00	43.10	43.70	44.40					
Lcc14	40.60	38.30	38.10	40.90	41.80	40.70	41.80	42.80	84.50				
Lcc9	42.20	40.90	40.40	42.70	43.20	43.00	43.50	45.20	87.00	84.20			
Lcc10	28.00	26.20	27.90	26.30	26.30	27.80	27.30	28.00	26.50	25.60	27.10		
Lcc11	25.80	27.00	25.60	27.20	24.60	26.70	27.40	26.30	27.80	26.40	27.90	23.60	

Lcc8 (Wong et al. 2013) was excluded because Lcc8 was not identified in D703PP-9 genome

### Structure of multicopper oxidase-encoding genes in *L. edodes*

**Fig. 2:**
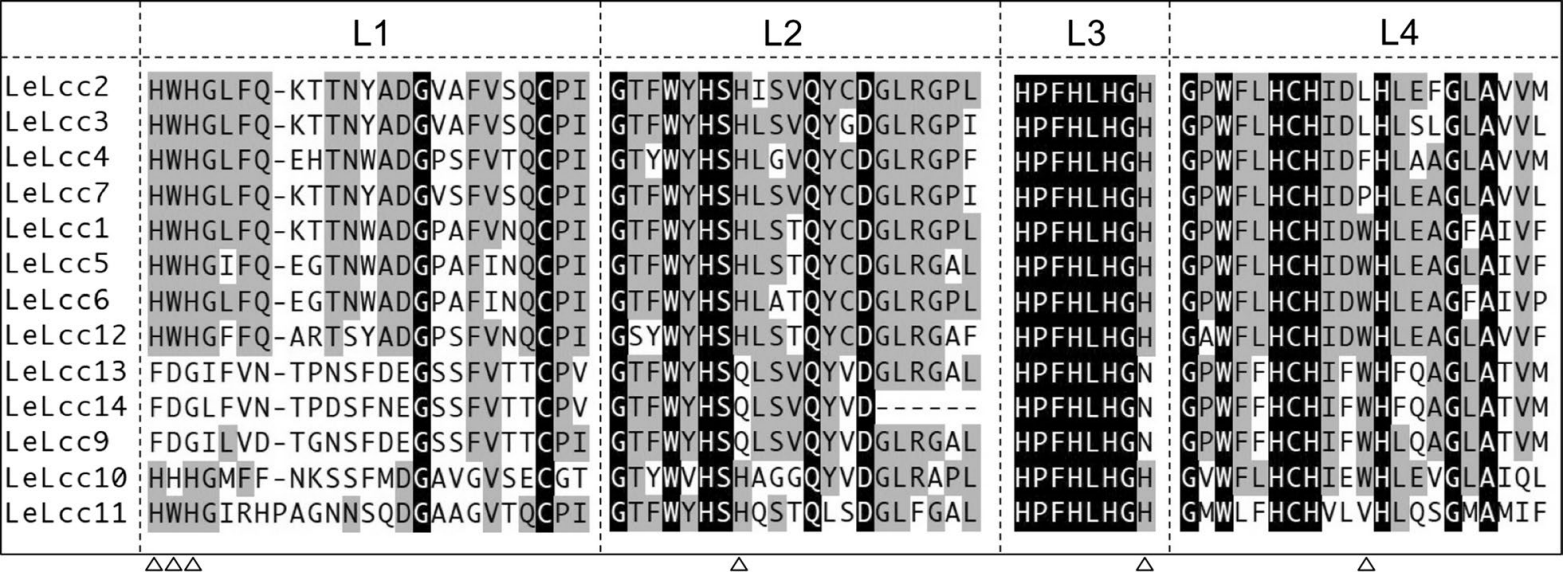
Signature sequences of laccases in *L. edodes. Black boxes* indicate perfectly conserved amino acids, and *gray boxes* indicate partially conserved amino acids. *Triangles* indicate characteristic differences among groups of multicopper oxidases in *L. edodes*

Analysis of alignments of multicopper oxidases-encoding genes suggested that all laccase encoding genes (laccase sensu stricto subfamily 1 and subfamily 2) had the 4 signature sequences (Fig. 2) defined by Kumar et al. (2003) and Kilaru et al. (2006). Two ferroxidases (Lcc10 and Lcc11) had low similarity to laccases sensu stricto (Table 2), but also had the 4 signature sequences (Fig. 2). Lower similarity was observed in multicopper oxidases in laccase sensu stricto subfamily 2, except for Lcc12, in signature 1 (L1). In Lcc9, Lcc13 and Lcc14 in laccase sensu stricto subfamily 2, the conserved histidines and the intermediate tryptophan (His-Trp-His) in the N-terminal copper binding element L1 were missing (indicated as triangles), and the conserved His in L2 has been changed to Gln, and the His in L3 to Asn in Lcc9, 13, and 14 (Fig. 2, indicated as triangles). Copper ion sites in laccases are classified into two types: type I (T1), where the electrons from the reducing substrates are accepted, and a type I/type III pair (T2/T3), which is assembled in a T2/T3 trinuclear cluster where electrons are transferred to perform the reduction of O_2_ to H_2_O (Ferraroni et al. 2007; Piontek et al. 2002). Lcc9, Lcc13 and Lcc14 each have the conserved histidines for T1 copper binding, but lack several T2/T3 copper binding histidines, suggesting different affinity to T2/T3 copper ions than laccase sensu stricto subfamily 1. We found a difference in amino acid sequence in two groups (group A: Lcc1, Lcc5 and Lcc6: group B: Lcc2, Lcc3, Lcc4 and Lcc7) in laccase sensu stricto subfamily 1, a tryptophan neighboring the copper binding conserved histidine in L4 (Fig. 2, indicated as a triangle). This presumably is involved in different substrate specificities of laccases in group A and group B in laccase sensu stricto subfamily 1.

Signal peptide analysis of multicopper oxidases showed that except for Lcc11 in *L. edodes*, these proteins have a putative signal peptide (D-cutoff = 0.450), and Lcc1, Lcc2, Lcc3, Lcc4, Lcc5, Lcc6 and Lcc10 have higher D-values than the other laccases (Additional file 1: Figure S1). Lcc1, Lcc2, Lcc3, Lcc4, Lcc5 and Lcc6 have been purified and their N-terminal amino acid sequences analyzed (Lcc1: Nagai et al. 2002; Lcc6: Nagai et al. 2009; Lcc2, Lcc4, Lcc5 and Lcc13: this study, Additional file 1: Table S3; Figure S2). The N-termini of Lcc1, Lcc5, Lcc6, and Lcc13 were identical to the putative N-terminal sequences indicated by SignalP; therefore, the Lcc1, Lcc5, Lcc6, and Lcc13 signal peptides will likely be recognized and mediate secretion. The N-termini of Lcc2 and Lcc4 could not be analyzed, as they were blocked (Additional file 1: Table S3), suggesting that N-termini of these laccases would not be digested as signal peptides.

**Fig. 3:**
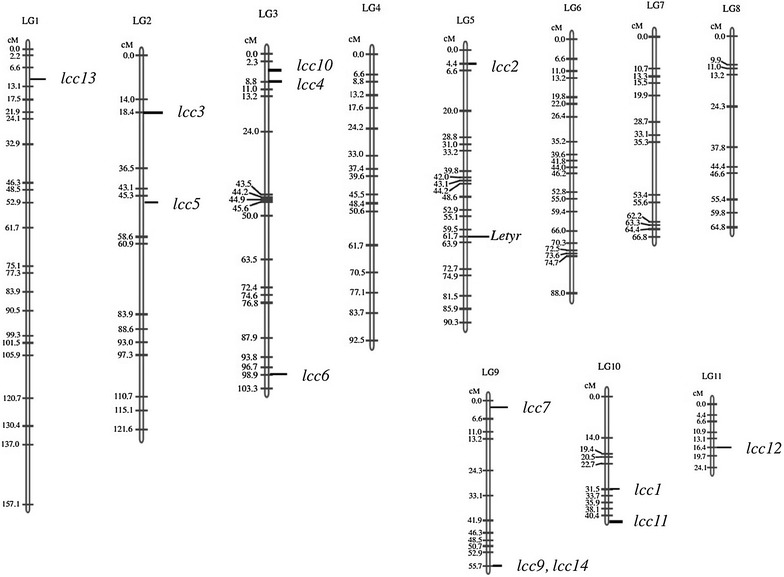
Linkage mapping of laccases in *L. edodes*. The genetic map was based on the linkage map constructed by Miyazaki et al. (2008) using an outbred line obtained by crossing a New Zealand strain (D703PP-9) and a Japanese strain (G408PP-4). The linkage group numbers, length of each linkage group and number of loci mapped on the linkage group are indicated on *top*. Distances (cM) between markers are shown on the *left* side. The names of the markers are shown on the *right* side

To analyze the distribution of laccase genes in the *L. edodes* genome, localization was carried out using a previously constructed linkage map of *L. edodes* (Miyazaki et al. 2008). The 13 multicopper oxidase-encoding genes were spread over 7 linkage groups. Comparably, in *P. ostreatus*, 12 laccases are located in 5 linkage groups (Castanera et al. 2012). No *L. edodes* laccases were located close together except for *lcc9* and *lcc14* (Fig. 3), in contrast to *C. cinerea,* in which four sets of laccases are located in tandem (Kilaru et al. 2006), or *P. ostreatus*, in which 7 laccase encoding genes are located on chromosome VI (Castanera et al. 2012).

### Transcription patterns of *L. edodes* multicopper oxidases

**Fig. 4:**
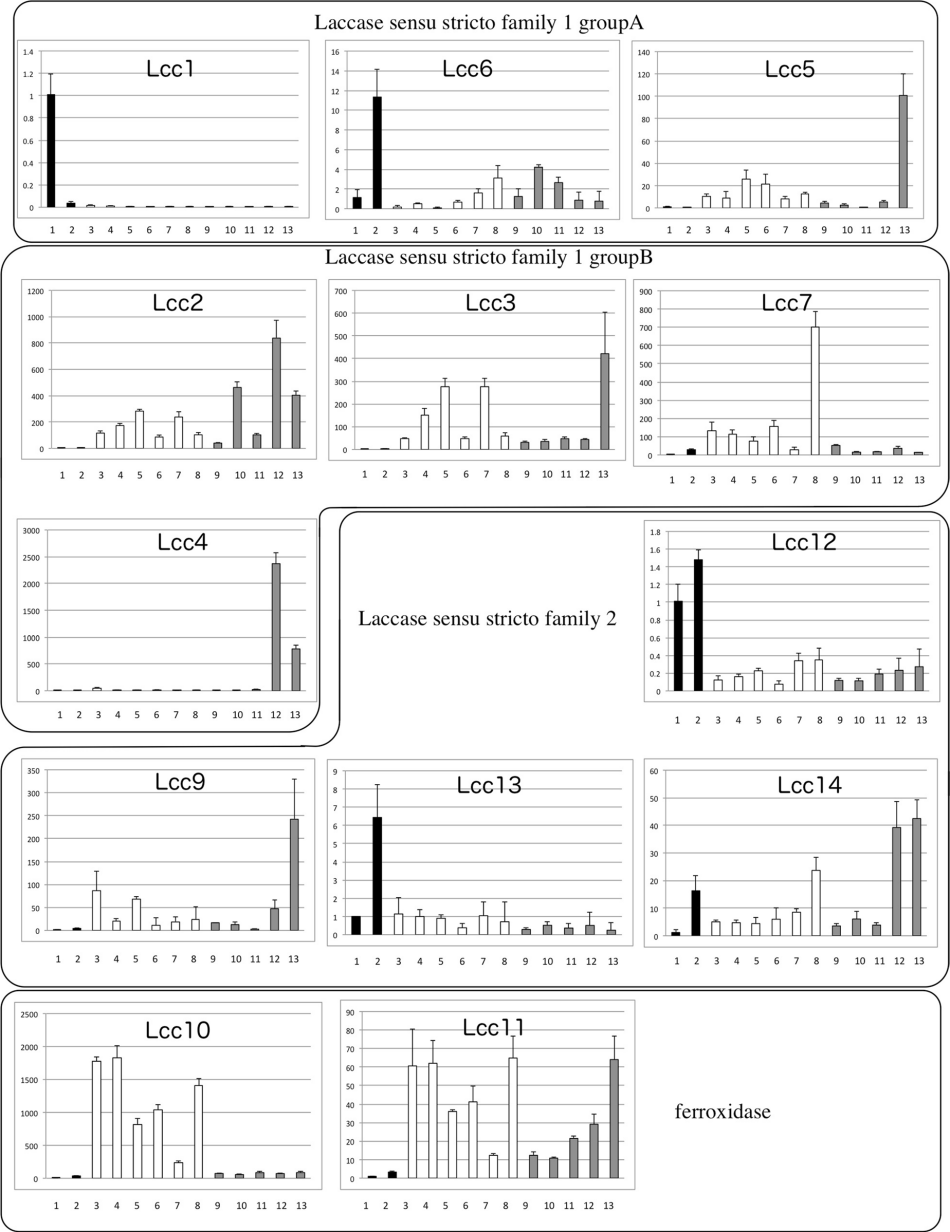
Transcription pattern of laccase encoding genes in *L. edodes*. Transcription levels of *lcc1* through *lcc7, lcc9* through *lcc14.* Y-axis means relative expression levels compared with expression levels of mycelia from liquid culture (*1*). All data points are mean ± SD (n = 3). *1* Mycelia from liquid culture. *2* Mycelia grown on sawdust medium. *3* Young fruiting bodies under 1 cm long. *4* Young fruiting bodies, 1–2 cm. *5* Stipe of young fruiting bodies, 2–3 cm. *6* Pileus of young fruiting bodies, 2–3 cm. *7* Stipe of young fruiting bodies, 3–5 cm. *8* Pileus of young fruiting bodies, 3–5 cm. *9* Gills of mature fruiting body; *10* gills of fruiting body 1 day after harvest; *11* gills of fruiting body 2 days after harvest; *12* gills of fruiting body 3 days after harvest; *13* gills of fruiting body 4 days after harvest. *Black Bar* indicates transcription in mycelia, *white bar* indicates transcription in growing fruiting body and *gray bar* indicates transcription in gills of fruiting body after harvest

Except for *lcc1* (Nagai et al. 2002; Sakamoto et al. 2008) and *lcc4* (Nagai et al. 2003; Sakamoto et al. 2009; Yano et al. 2009), the expression patterns of multicopper oxidase-encoding genes in *L. edodes* have not been well characterized. Using real-time PCR (Fig. 4) and calculation of expression as the ratio relative to expression in mycelia grown in liquid culture, we compared expression patterns of individual multicopper oxidases in *L. edodes*.

In laccase sensu stricto subfamily 1, *lcc1* was more highly transcibed in mycelia than in fruiting bodies (Fig. 4), in agreement with previous reports (Sakamoto et al. 2008), The corresponding protein Lcc1 was secreted into liquid culture (Nagai et al. 2002) and sawdust media (Additional file 1: Figure S3). *lcc6* was transcribed in mycelia grown on sawdust media (Fig. 4, Additional file 1: Figure S3 and as shown in Nagai et al. 2009), and also transcribed in fruiting bodies (Fig. 4). *lcc5* was expressed in fruiting bodies after harvest, but was transcribed at low levels in liquid culture medium and in mycelia cultured on sawdust media as a colony. However, Lcc5 was found to be secreted in the outer edges of the colonies (Additional file 1: Figure S3; Nagai et al. 2009).

Transcription of *lcc2, lcc3*, *lcc4* and *lcc7* (laccase sensu stricto subfamily 1) was low in mycelia grown in liquid medium or on sawdust medium (Fig. 4). Genes *lcc2* and *lcc3* were transcribed in the fruiting body, transcription increased during growth of the fruiting body, and transcription was higher in the pileus than in the stipe. Transcription of *lcc2* also increased in the gills immediately after harvesting of the fruiting body (Fig. 4, Additional file 1: Figure S5). However, transcription of *lcc3* in gills was very low during post-harvest preservation. Western blotting confirmed the presence of Lcc2 and Lcc3 in fruiting bodies, especially in the pileus (Additional file 1: Figure S4). Gene *lcc4* was specifically transcribed in gills after harvesting of the fruiting body (Fig. 4, Additional file 1: Figure S5) at days 3 and 4 after harvest, and was transcribed more slowly than *lcc2* (in case of *lcc2,* transcribed from day2 after harvest). Gene *l*cc7 was expressed specifically in fruiting bodies, but transcription was lower after harvest than in fresh fruiting bodies. These observations suggest that members of laccase sensu stricto subfamily 1 group B are mainly transcribed in fruiting bodies.

The laccases in sensu stricto subfamily 2, *lcc9*, *lcc12, lcc13,* and *lcc14*, showed varying transcription in mycelia and fruiting bodies. Genes *lcc12*, *lcc13* and *lcc14* were transcribed in mycelia grown on sawdust medium while *lcc9* was not or was transcribed at low levels. Transcription of *lcc14* was also elevated in fruiting bodies after harvest. Gene *lcc9* was mainly expressed in fruiting bodies after harvest.

The ferroxidases, *lcc10* and *lcc11*, were expressed specifically in fruiting bodies (Fig. 4). Gene *lcc10* was expressed most strongly in fresh fruiting bodies, while *lcc11* was expressed in fresh fruiting bodies but showed an increase in expression after harvest. The two genes were not transcribed in mycelia, suggesting that these two genes are fruiting body specific.

## Discussion

Previously, 11 laccase encoding genes (*lcc1* through *lcc11*) were reported for *L. edodes* (Additional file 1: Table S6, Wong et al. 2013). In this paper, we identified three new laccase genes in *L. edodes* D703PP-9 (*lcc12*–*14*). Subsequent analysis showed that products of genes *lcc10* and *lcc11* (Wong et al. 2013) are actually not laccases sensu stricto but are ferroxidases instead, bringing the total to 11 laccases sensu stricto in D703PP-9. Phylogenetic mapping of the 13 multicopper oxidases in *L. edodes* showed that they can be classified into laccase sensu stricto subfamily 1, sensu stricto subfamily 2, and ferroxidases (Fig. 1; Table 2). On the other hand, we could not identify *lcc8* (Wong et al. 2013) in D703PP-9. This would depend on far genetic distance of D703PP-9 from Asian *L. edodes* strains (Miyazaki and Neda 2004). We also could not amplify *lcc8* from H600 cDNAs (data not shown). Therefore, further genomic comparison among several *L. edodes* strains is needed to reveal the exact number of multicopper oxidases in *L. edodes* genome.

We investigated expression patterns of multicopper oxidases in three stages of the *L. edodes* life cycle, mycelia, growing fruiting bodies, and fruiting bodies after harvest (Fig. 4). We found that *lcc1, lcc6, lcc5* (laccase sensu stricto subfamily 1 group A), and *lcc12, lcc13,* and *lcc14* (laccase sensu stricto subfamily 2) were expressed in mycelia (Fig. 4), and Lcc1, Lcc6, Lcc5 and Lcc13 are found to be secreted into sawdust medium (Additional file 1: Figure S2; Table S3, Nagai et al. 2002, 2009). In contrast to multicopper oxidases expressed in mycelia, almost all multicopper oxidases are expressed in *L. edodes* fruiting bodies. Multicopper oxidases in laccase sensu stricto subfamily 1 group B (*lcc2, lcc 3, lcc 4* and *lcc 7*) and ferroxidases (*lcc10* and *lcc11*) were expressed mainly in fruiting bodies but transcribed at low levels or not at all in mycelia (Fig. 4). These observation suggest that laccases in same group in *L. edodes* share expression patters. On the other hand, we earlier revealed that many genes involved in senescence of fruiting bodies of *L. edodes* are expressed after harvest (Sakamoto et al. 2009, 2012). Some multicopper oxidases transcribed after harvest must be involved in browning of fruiting bodies after harvest coordinately with tyrosinase (Nagai et al. 2003; Sakamoto et al. 2009, 2012; Sato et al. 2009). Many multicopper oxidases of all groups, *lcc2, lcc3, lcc4, lcc5, lcc9, lcc11,* and *lcc14*, were transcribed in fruiting bodies after harvest. This observation suggests that global transcription changes for multicopper oxidase-encoding genes occur at the stage of senescence.

Some of the laccases that share sequence similarities possibly have similar biological functions. We found that *L. edodes* laccases in sensu stricto subfamily 1 can be divided into two major groups by sequence similarities and expression patterns, one is group of laccases secreted from mycelia (*lcc1, lcc5, lcc6*: group A) and the other is a group of laccases mainly expressed in fruiting body (*lcc2, lcc3, lcc4* and *lcc7*: group B). In laccase sensu stricto subfamily 1 group A, Lcc1 and Lcc6 are closely related (Fig. 1). Both of the two laccases are expressed in cultivated mycelia and are secreted (Fig. 4, Additional file 1: Figure S3). We compared multicopper oxidases in *Marasmiaceae* species [*L. edodes*, *O. olearius* (Wawrzyn et al. 2012), *D. bispora*: http://genome.jgi.doe.gov/Denbi1/Denbi1.home.html*, M. perniciosa* (Mondego et al. 2008) and *G. luxurians* (Kohler et al. 2015)], for which public genome sequences are available, and only *G. luxurians* has multiple laccases closely related to Lcc1 and Lcc6. On the other hand, Lcc5, which has higher similarity to Lcc1 and Lcc6, is clustered in a different clade in the phylogenetic tree (Fig. 1), but clusters with laccases in all *Marasmiaceae* species tested, except for the pathogenic fungus *M. perniciosa.* Lcc5 is expressed and secreted into sawdust media similarly to *lcc6*, suggesting that Lcc5 and Lcc6 may have a similar biological function in hyphal growth in sawdust media. Laccases are presumably involved in lignin degradation by white-rot fungi (Baldrian 2006; Eggert et al. 1997; Rivera-Hoyos et al. 2013; Thurston 1994); therefore, laccase secreted from mycelia grown on sawdust medium in laccase sensu stricto subfamily 1 group A (*lcc1, lcc5* and *lcc6*) may be involved in lignin degradation as well. Only the pathogenic fungus *M. perniciosa* does not have these laccases, suggesting that *M. perniciosa*, which does not rely on rotting wood for growth, might not need this type of laccase. On the other hand, Lcc1 is involved in hyphal morphogenesis and cell wall synthesis in *L. edodes* (Nakade et al. 2011). Furthermore, several research groups have reported that laccase activity is high in colonized mycelium and decreases during fruiting body development (Elisashvili et al. 2008; Ohga et al. 1998, 2000; Ohga and Royse 2001; Kües and Liu 2000). It has also been reported that phenoloxidase (including laccase) activity correlates with fruiting body formation in mushrooms (Leonard and Phillips 1973; Suguimoto et al. 2001; Vnenchak and Schwalb 1989). Furthermore, Magae et al. (2005) suggested that Bromophenol Blue decolorization ability correlates with fruiting body formation, and Lcc1 in *L. edodes* has this ability. More recently, overexpression of laccase in *Hypsizygus marmoreus* enhances fruiting body production (Zhang et al. 2015). These observations collectively suggest that Lcc1 in *L. edodes* may be correlated with fruiting body formation (Nakade et al. 2011). Therefore, further studies are needed to reveal the relationship between these secreted types of laccases in sensu stricto subfamily 1 group A and hyphal development or fruiting body formation.

Laccases in another group of laccase sensu stricto subfamily 1, groupB: *lcc2, lcc3, lcc4* and *lcc7*, are mainly expressed in fruiting bodies. All *Marasmiaceae* species tested have this type of laccase (Fig. 1). This suggests that laccases in sensu stricto subfamily 1 group B could have common biological roles in *Marasmiaceae* species. *lcc2,lcc3* and *lcc7* are transcribed in fresh fruiting bodies, and *lcc2*, *lcc3* and *lcc4* are transcribed in fruiting bodies after harvest (Fig. 4, Additional file 1: Figures S4, S5). This suggests that these laccases are performing their main role in fruiting bodies. This is supported by other findings; for example, Lcc4 can catalyze the oxidization of L-DOPA, and is involved in melanin synthesis (gill browning), but Lcc1 and Lcc6 cannot (Nagai et al. 2003, 2009) or are weak catalysts compared to Lcc4 (Nagai et al. 2003; Sakamoto et al. 2012; Wong et al. 2013). To clarify the classification and biological functions of the laccase sensu stricto subfamily 1 group B in *Marasmiaceae* species, further enzymatic investigations in other laccases of this subfamily are needed.

*Marasmiaceae* species also have two different types of laccases in sensu stricto subfamily 2. One is a group including Lcc12 and the other is a group including Lcc9, Lcc13 and Lcc14. Interestingly, laccase sensu stricto subfamily 2 is generally conserved in limited species of the *Agaricales*, such as *L. edodes*, *F. velutipes, C. cinerea, A. bisporus* and *P. ostreatus* (Fig. 1). There is little information on laccase sensu stricto subfamily 2 in *L. edodes.* Furthermore, enzymatic characteristics and biological functions of laccases in this subfamily have not been well characterized. *lcc13* is transcribed fairly abundantly in sawdust medium (Fig. 4) and Lcc13 secreted into the medium (Additional file 1: Figure S2). Three of four laccases in sensu stricto subfamily 2 in *L. edodes* are transcribed in mycelia and have a signal peptide for secretion; therefore, laccases in sensu stricto subfamily 2 are likely to function as extracellular enzymes for lignin degradation.

Wong et al. (2013) reported that Lcc10 and Lcc11 in *L. edodes* can be classified as laccases, but our data suggests that these two laccases could be categorized as ferroxidases following the classification of multicopper oxidases according to Hoegger et al. (2006) and Kües and Rühl (2011). Lcc10 is classified as a fungal ferroxidase (Fet3-type) conserved in basidiomycetes (Fig. 1, Kües and Rühl 2011). *G. luxurians* and *O. olearius* of the *Marasmiaceae* tested have a similar type of multicopper oxidase. Lcc11 is classified as a ferroxidase/laccase conserved in a wide range of basidiomycetes and ascomycetes, but only *G. luxurians* of the *Marasmiaceae* tested has a similar type of multicopper oxidase. These ferroxidases are specifically expressed in *L. edodes* fruiting bodies, but further studies are needed to determine their biological functions.

In conclusion, we found 13 distinct multicopper oxidases in the *L. edodes* D703PP-9 genome. These multicopper oxidases can be classified by sequence similarities and differentiated functionally, like chitin synthase in *Yarrowia lipolytica* (Sheng et al. 2013). The 1000 Fungal Genomes Project provided large amounts of genome data for basidiomycetous fungi, and wood decay mechanisms are discussed based on plant cell wall degradation related enzymes, such as lignin and cellulose degradation enzymes (Floudas et al. 2012, 2015; Riley et al. 2014). Relationship between evolution of lignin peroxidases and basidiomycetous fungi has been well discussed in the papers. More laccases are found in basidiomycetous fungal genomes compare with fungal peroxidases (Floudas et al. 2012). Therefore, biological function of laccase could be more diverse. Further enzymatic study and functional analysis will provide insights into how this multiplicity of laccases evolved and became functionally differentiated for lignin degradation, fruiting body development and fruiting body coloration.

## Additional file

**Additional file 1.** Additional information of *L. edodes* multicopper oxidases, such as signal peptide prediction, enzyme purificaition, expression patterns, primer design, data set for phylogenetic analysis, and sequence infromation.
