# Supplementary material for: Grouping of multicopper oxidases in Lentinula edodes by sequence similarities and expression patterns
Source: AMB Express. 2015 Sep 17;5:63. doi: 10.1186/s13568-015-0151-2 (PMC4573974; doi:10.1186/s13568-015-0151-2)
Supplement: Supplementary file 1 — Additional file 1. Additional information of L. edodes multicopper oxidases, such as signal peptide prediction, enzyme purificaition, expression patterns, primer design, data set for phylogenetic analysis, and sequence infromation. [file 13568_2015_151_MOESM1_ESM.pdf]

Journal name: AMB express

Manuscript Title: **Grouping of multicopper oxidases in *Lentinula edodes* by sequence similarities and expression patterns.**

Names of Authors: Yuichi Sakamoto[a], Keiko Nakade[a, b], Kentaro Yoshida[a, c], Satoshi Natsume[a], Kazuhiro Miyazaki[d], Shiho Sato[a], Arend F. van Peer[a, e], Naotake Konno[a, f]

[a]Iwate Biotechnology Research Center, Japan
[b]TSUMURA and Co., 3586 Yoshiwara, Ami-machi Inashiki-gun, Ibaraki, 300-1192, Japan,
[c] Graduate School of Agricultural Science, Kobe University, 1-1 Rokkodai, Nada-ku, Kobe, 657-8501, Japan
[d]Kyushu Research Center Forestry and Forest Products Research Institute, 4-11-16 Kurokami, Kumamoto, Kumamoto, 860-0862, Japan,
[e]College of Life Sciences, Fujian Agriculture and Forestry University, Fuzhou 350002, PR China
[f]Utsunomiya University, Faculty of Agriculture, 350 Mine-machi, Utsunomiya, Tochigi 321-8505, Japan

Corresponding author: Yuichi Sakamoto
Mailing address: Iwate Biotechnology Research Center, 22-174-4 Narita, Kitakami-shi, Iwate, 024-0003 Japan.

Tel: +81-197-68-2911. Fax: +81-197-68-3881. E-mail: sakamoto@ibrc.or.jp

Lcc1

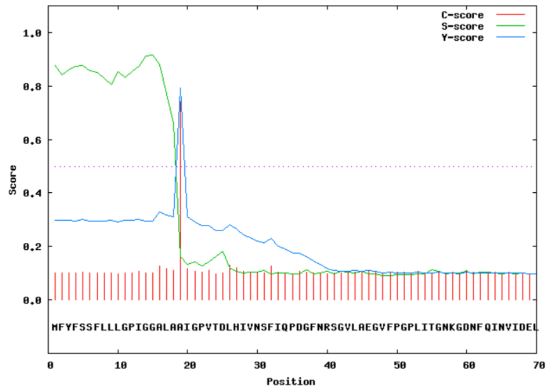

# Measure	Position	Value	Cutoff	signal peptide?
max. C	19	0.740		
max. Y	19	0.794		
max. S	15	0.916		
mean S	1-18	0.846		
D	1-18	0.822	0.450	YES

Name=LeLcc1 SP='YES' Cleavage site between pos. 18 and 19: ALA-AI D=0.822 D-cutoff=0.450

Lcc2

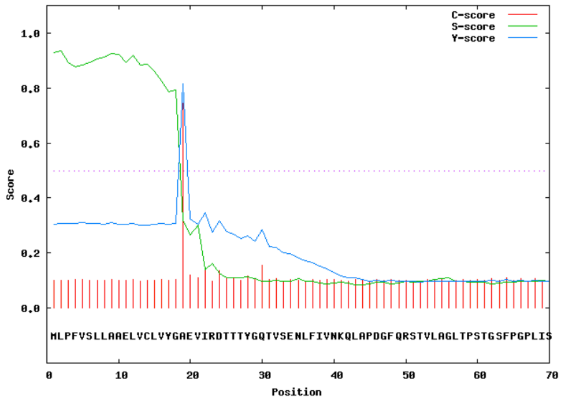

# Measure	Position	Value	Cutoff	signal peptide?
max. C	19	0.743		
max. Y	19	0.814		
max. S	2	0.936		
mean S	1-18	0.885		
D	1-18	0.852	0.450	YES

Name=LeLcc2 SP='YES' Cleavage site between pos. 18 and 19: VYG-AE D=0.852 D-cutoff=0.450

Lcc3

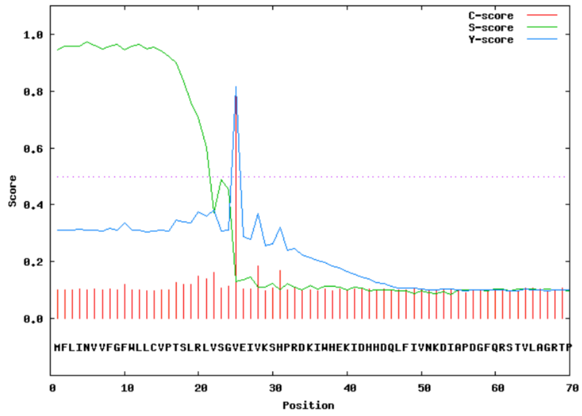

# Measure	Position	Value	Cutoff	signal peptide?
max. C	25	0.791		
max. Y	25	0.815		
max. S	5	0.974		
mean S	1-24	0.849		
D	1-24	0.833	0.450	YES

Name=LeLcc3 SP='YES' Cleavage site between pos. 24 and 25: VSG-VE D=0.833 D-cutoff=0.450

Lcc4

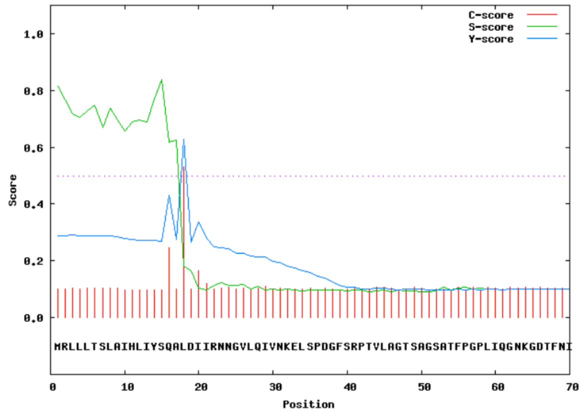

# Measure	Position	Value	Cutoff	signal peptide?
max. C	18	0.532		
max. Y	18	0.627		
max. S	15	0.837		
mean S	1-17	0.716		
D	1-17	0.675	0.450	YES

Name=LeLcc4 SP='YES' Cleavage site between pos. 17 and 18: SQA-LD D=0.675 D-cutoff=0.450

Lcc5

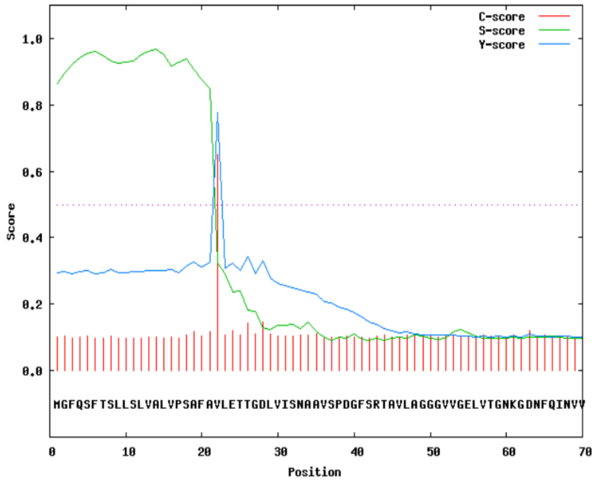

# Measure	Position	Value	Cutoff	signal peptide?
max. C	22	0.652		
max. Y	22	0.776		
max. S	14	0.969		
mean S	1-21	0.926		
D	1-21	0.857	0.450	YES

Name=LeLcc5 SP='YES' Cleavage site between pos. 21 and 22: AFA-VL D=0.857 D-cutoff=0.450

Lcc6

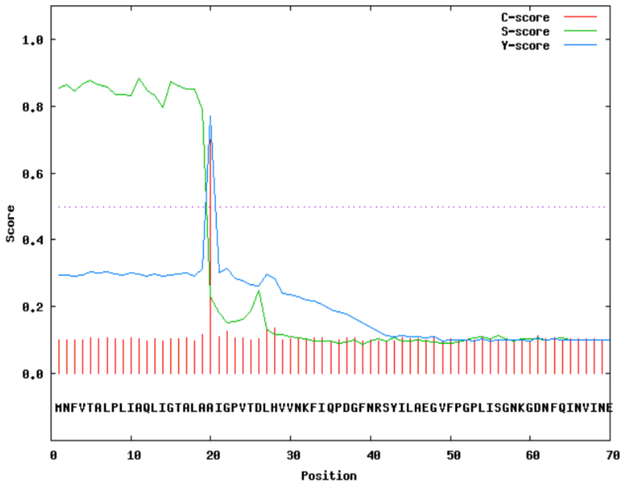

# Measure	Position	Value	Cutoff	signal peptide?
max. C	20	0.699		
max. Y	20	0.770		
max. S	11	0.882		
mean S	1-19	0.848		
D	1-19	0.812	0.450	YES

Name=LeLcc6 SP='YES' Cleavage site between pos. 19 and 20: ALA-AI D=0.812 D-cutoff=0.450

Lcc7

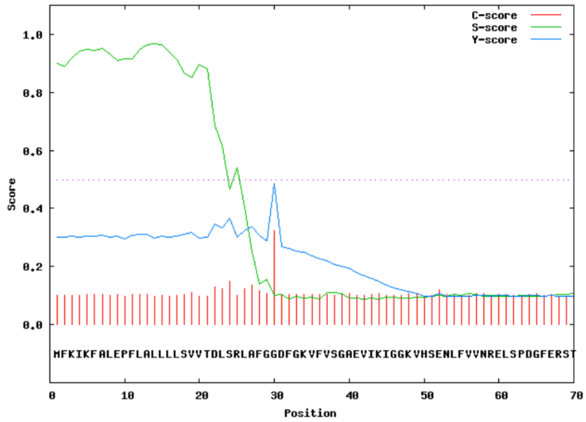


```
# Measure  Position  Value    Cutoff   signal peptide?
  max. C    30       0.324
  max. Y    30       0.487
  max. S    14       0.967
  mean S     1-29    0.780
       D     1-29    0.645   0.450    YES
Name=LeLcc7     SP='YES' Cleavage site between pos. 29 and 30: AFG-GD D=0.645 D-cutoff=0.450
```

Lcc9

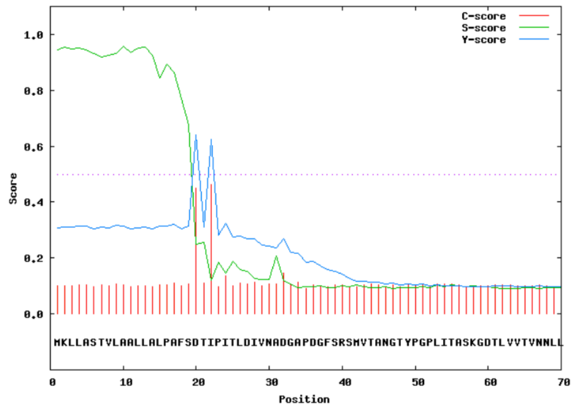


```
# Measure  Position  Value    Cutoff   signal peptide?
  max. C    22       0.462
  max. Y    20       0.640
  max. S    10       0.958
  mean S     1-19    0.906
       D     1-19    0.784   0.450    YES
Name=LeLcc11    SP='YES' Cleavage site between pos. 19 and 20: AFS-DT D=0.784 D-cutoff=0.450
```

Lcc10

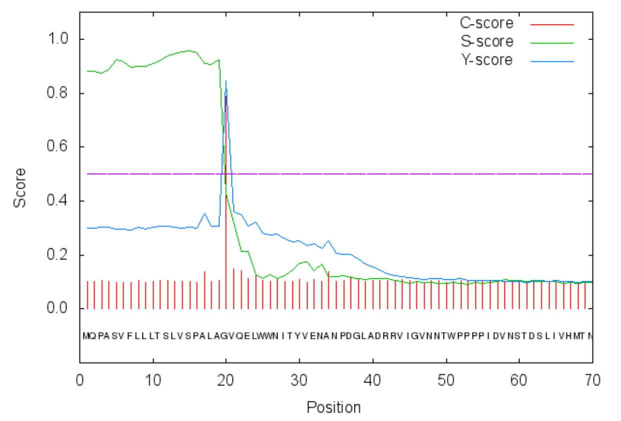


```
# Measure  Position  Value    Cutoff   signal peptide?
  max. C    20       0.786
  max. Y    20       0.845
  max. S    15       0.955
  mean S     1-19    0.914
       D     1-19    0.883   0.450    YES
Name=Sequence   SP='YES' Cleavage site between pos. 19 and 20: ALA-GV D=0.883 D-cutoff=0.450
```

Lcc11

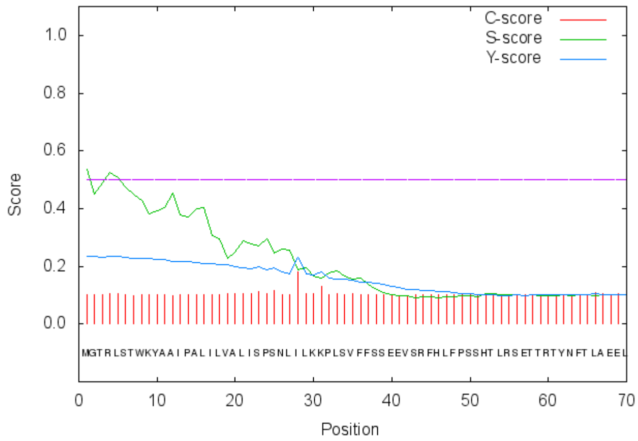


```
# Measure  Position  Value    Cutoff   signal peptide?
  max. C    28       0.182
  max. Y    28       0.232
  max. S     1       0.536
  mean S     1-27    0.371
       D     1-27    0.288   0.500    NO
Name=Sequence   SP='NO' D=0.288 D-cutoff=0.500 Networks=SignalP-TM
```

Lcc12

>LeLcc8

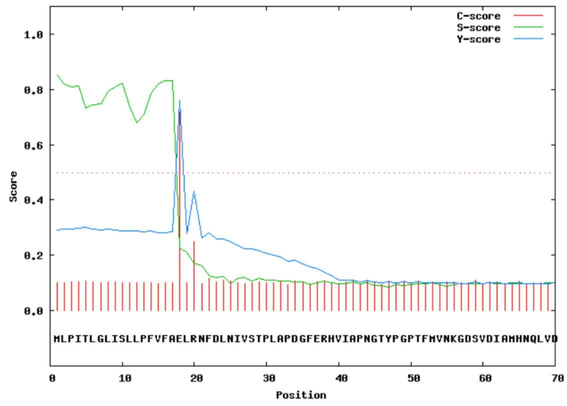


```
# Measure  Position  Value    Cutoff   signal peptide?
  max. C    18       0.720
  max. Y    18       0.759
  max. S     1       0.852
  mean S     1-17    0.784
       D     1-17    0.773   0.450    YES
Name=LeLcc8     SP='YES' Cleavage site between pos. 17 and 18: VFA-EL D=0.773 D-cutoff=0.450
```

Lcc13

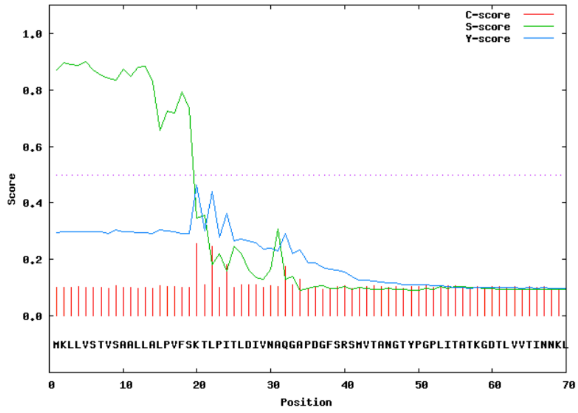


```
# Measure  Position  Value    Cutoff   signal peptide?
  max. C    20       0.255
  max. Y    20       0.462
  max. S     5       0.901
  mean S     1-19    0.831
       D     1-19    0.661   0.450    YES
Name=LeLcc9     SP='YES' Cleavage site between pos. 19 and 20: VFS-KT D=0.661 D-cutoff=0.450
```

Lcc14

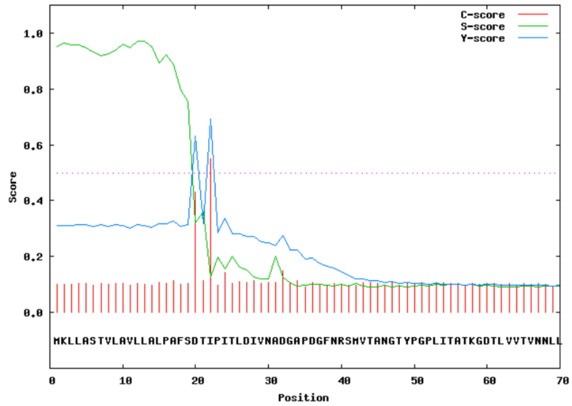


```
# Measure  Position  Value    Cutoff   signal peptide?
  max. C    22        0.551
  max. Y    22        0.693
  max. S    13        0.971
  mean S     1-21     0.868
       D     1-21     0.787   0.450   YES
Name=LeLcc10    SP='YES' Cleavage site between pos. 21 and 22: SDT-IP D=0.787 D-cutoff=0.450
```

Fig. S1 Analysis of signal peptides of Lcc1 through Lcc7, Lcc9, and Lcc12 through Lcc14. Putative signal peptides were predicted by SignalP (http://www.cbs.dtu.dk/services/SignalP/).

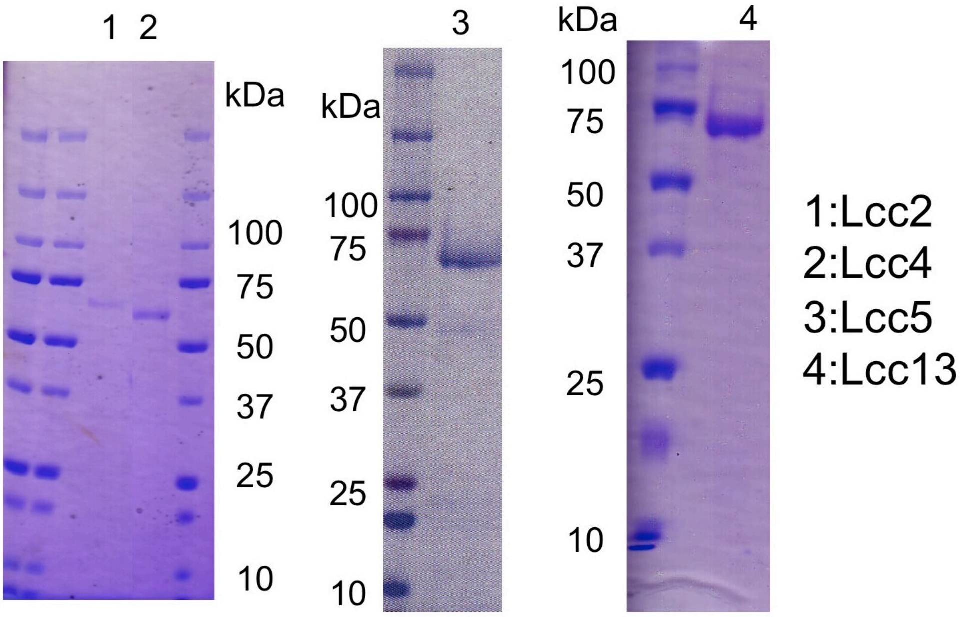

Fig. S2 Electrophoresis of purified laccases, Lcc2, Lcc4, Lcc5 and Lcc13. Purification steps are described in Table S1.

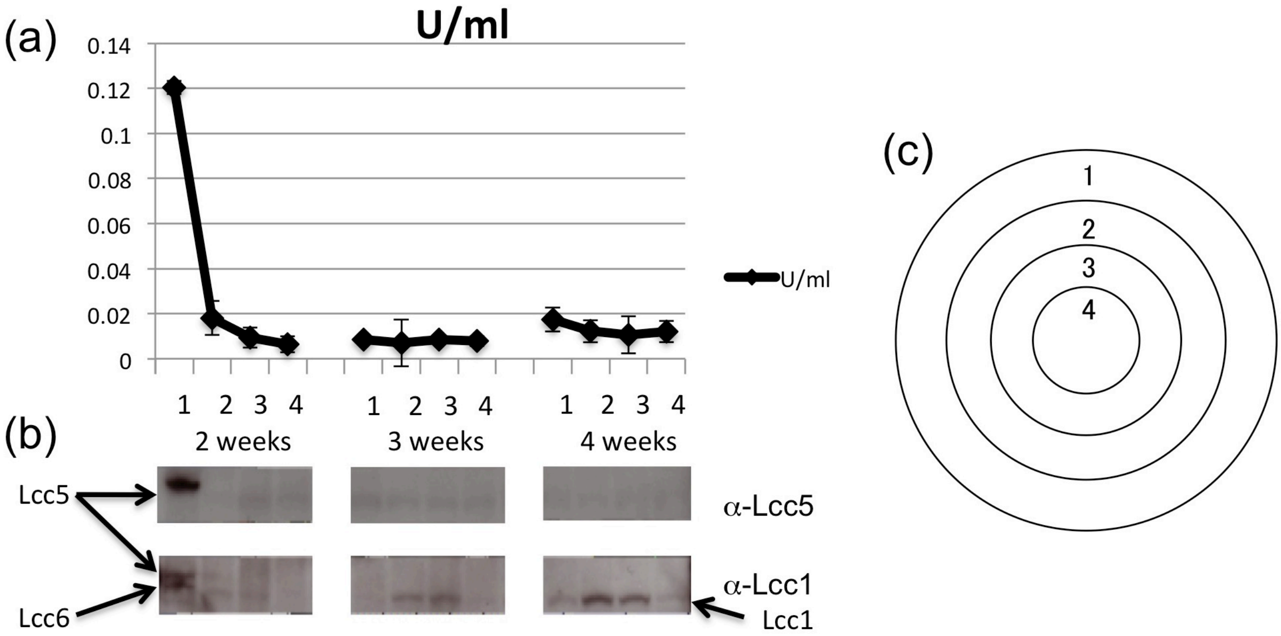

Fig. S3 Laccase activity and secretion of Lcc5 and Lcc6 on sawdust medium. (a) laccase activity on sawdust medium inoculated after 2 weeks, 3 weeks, ad 4 weeks. (b) Western blot analysis of Lcc5 and Lcc6 secreted into sawdust medium using α- Lcc5 (upper panel) and α- Lcc1 (lower panel), respectively. (c) position of sawdust medium in the petri dish for enzyme extraction.

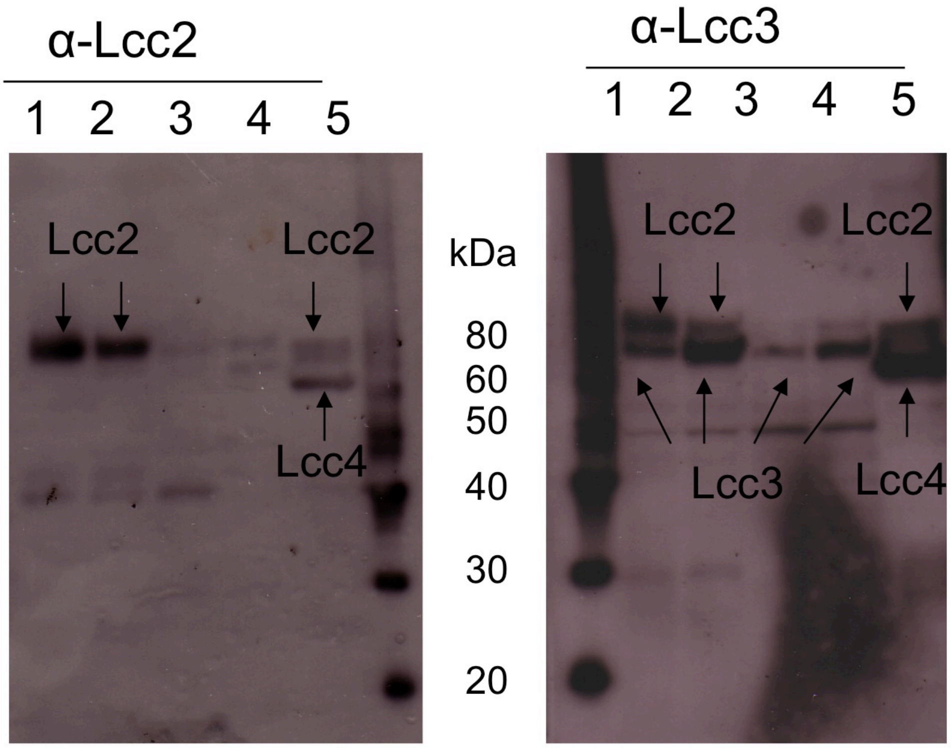

Fig. S4 Western blot analysis of enzymes extracted from several fruiting body tissues using Lcc2 and Lcc3 anti-serum. Crude enzymes from 1: pileus peel, 2: pileus (without peel), 3: stipe, 4: gills, 5: gills 4 days after harvest.

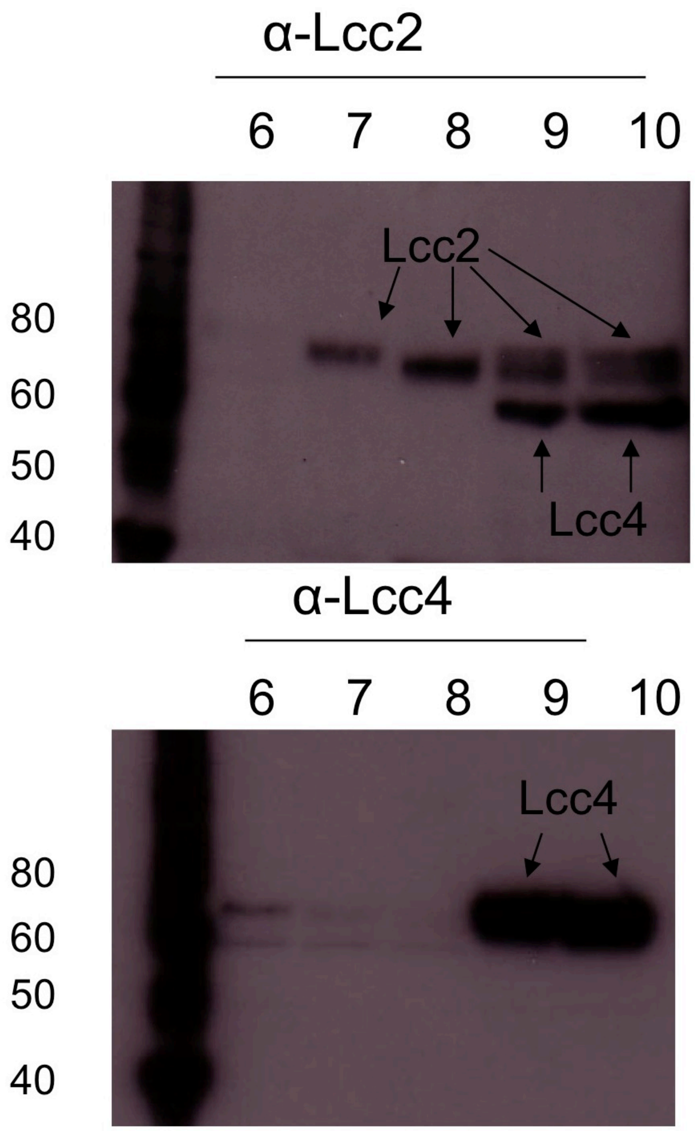

Fig. S5 Western blot analysis of enzymes extracted from fruiting bodies after harvest using Lcc4 anti-serum. 6: gills of fresh fruiting bodies, 7: gills of fruiting bodies 1 day after harvest, 8: gills of fruiting bodies 2 days after harvest, 9: gills of fruiting bodies 3 days after harvest, 10: gills of fruiting body 4 days after harvest.

Table S1 Primers used in ORF amplification

primer_name	sequence
Lcc7_ATGU	ATGTTCAAGATCAAATTTGCTCTCG
Lcc7_TGAL	TCAAGGCAGTTGATCAGGAGTCAAA
Lcc9_ATGU	ATGAAACTCCTCGCTTCTACCGTT
Lcc9_TGAL	TCACTGCAAATCAGGTGAAAG
Lcc12_ATGU	ATGTTGTCCATCACACTCGGCCT
Lcc12_TGAL	CTACTGCAATTCGGGTTCCAGAG
Lcc13_ATGU	ATGAAGCTCCTCGTTTCTACTGT
Lcc13_TAAL	CGCTCTTCCTGCTGACTTACAATAA
Lcc14_ATGU	ATGAAGCTCCTCGCGTCTAC
Lcc14_TGAL	TCACTGTAGATCAGCTGGA

Table S2 Accession numbers of laccase encoding genes in Fig. 1

gene name	accession no.	species
CcLcc11	BK004121	*Coprinopsis cinerea*
CcLcc12	BK004122	*Coprinopsis cinerea*
CcLcc14	BK004124	*Coprinopsis cinerea*
CcLcc16	BK004126	*Coprinopsis cinerea*
CcLcc17	BK004127	*Coprinopsis cinerea*
CcLcc1	BK004111	*Coprinopsis cinerea*
CcLcc2	BK004112	*Coprinopsis cinerea*
CcLcc3	BK004113	*Coprinopsis cinerea*
CcLcc4	BK004114	*Coprinopsis cinerea*
CcLcc5	BK004115	*Coprinopsis cinerea*
CcLcc6	BK004116	*Coprinopsis cinerea*
CcLcc7	BK004117	*Coprinopsis cinerea*
CcLcc9	BK004119	*Coprinopsis cinerea*
LeLcc1	AB035409	*Lentinula edodes*
LeLcc2	AB035410	*Lentinula edodes*
LeLcc3	AB046713	*Lentinula edodes*
LeLcc4	AB446445	*Lentinula edodes*
LeLcc5	AB543788	*Lentinula edodes*
LeLcc6	AB543787	*Lentinula edodes*
LccK	AB089612	*pleurotus ostreatus*
PbLac1	EF362634	*Polyporus brumalis*
FvLcc2	AB252575	*Flammulina velutipes*
LeLcc7	AB821483	*Lentinula edodes*
LeLcc12	AB821484	*Lentinula edodes*
LeLcc13	AB821485	*Lentinula edodes*
LeLcc14	AB821486	*Lentinula edodes*
LeLcc9	AB821487	*Lentinula edodes*
LeLcc8	JX879732	*Lentinula edodes*
LBgi_224472740	ACN49095.1	*Laccaria bicolor*
LBgi_224472736	ACN49093.1	*Laccaria bicolor*
LBgi_224472732	ACN49091.1	*Laccaria bicolor*
LBgi_224472728	ACN49089.1	*Laccaria bicolor*
LBgi_224472742	ACN49096.1	*Laccaria bicolor*
LBgi_224472738	ACN49094.1	*Laccaria bicolor*
LBgi_224472734	ACN49092.1	*Laccaria bicolor*
LBgi_224472730	ACN49090.1	*Laccaria bicolor*
LBgi_224472726	ACN49088.1	*Laccaria bicolor*
Polacc1	e_gw1.06.955.1	*pleurotus ostreatus*
Polacc2	estExt_Genewise1Plus.C_080562	*pleurotus ostreatus*
Polacc4	fgenesh1_pm.06_#_14	*pleurotus ostreatus*
Polacc5	estExt_fgenesh1_pm.C_110168	*pleurotus ostreatus*
Polacc6	estExt_Genemark1.C_060050	*pleurotus ostreatus*
Polacc7	fgenesh1_pm.06_#_154	*pleurotus ostreatus*
Polacc8	gm1.8462_g	*pleurotus ostreatus*
Polacc9	estExt_fgenesh1_kg.C_060016	*pleurotus ostreatus*
Polacc10	stExt_fgenesh1_kg.C_060005	*pleurotus ostreatus*
Scgi_3273348	BAA31217.1	*Schizophyllum commune*
Scgi_300102852	EFI94259.1	*Schizophyllum commune*
Scgi_302691486	XP_003035422.1	*Schizophyllum commune*
Scgi_302672793	XP_003026084.1	*Schizophyllum commune*
Tvgi_4218524	CAA77015.1	*Trametes versicolor*
Tvgi_392569192	EIW62366.1	*Trametes versicolor*

Tvgi_2598857	BAA23284.1	*Trametes versicolor*
Tvgi_390980641	AFM31222.1	*Trametes versicolor*
Tvgi_886719	CAA59161.1	*Trametes versicolor*
Tvgi_298239752	ADI70681.1	*Trametes versicolor*
Tvgi_195971815	ACG61163.1	*Trametes versicolor*
Tvgi_2388517	BAA22153.1	*Trametes versicolor*
LeLcc10	AGO04565	*Lentinula edodes*
LeLcc11	AGO04566	*Lentinula edodes*
Pp_Lcc1	refXP_002472930.1	*Postia placenta*
Pp_Lcc3	refXP_002475824.1	*Postia placenta*
Pp_Lcc4	refXP_002471087.1	*Postia placenta*
Aslcc	embCAC59820.1	*Aspergillus nidulans*
Ab_426192758	EKV42693.1	*Agaricus bisporus*
Ab_256002912	ACU52699.1	*Agaricus bisporus*
Ab_426198838	EKV48764.1	*Agaricus bisporus*
Ab_426196258	EKV46187.1	*Agaricus bisporus*
Ab_426194916	EKV44847.1	*Agaricus bisporus*
Ab_426198836	EKV48762.1	*Agaricus bisporus*
Ab_426192759	EKV42694.1	*Agaricus bisporus*
Ab_426198800	EKV48726.1	*Agaricus bisporus*
Ab_426196257	EKV46186.1	*Agaricus bisporus*
Ab_426194937	EKV44868.1	*Agaricus bisporus*
MCO_1	GSTUMT00002907001	*Tuber melanosporum*
MCO_2	GSTUMT00008992001	*Tuber melanosporum*
MCO_3	GSTUMT00006911001	*Tuber melanosporum*
MCO_4	GSTUMT00002988001	*Tuber melanosporum*
MCO_5	GSTUMT00009952001	*Tuber melanosporum*
Polar1_502186	e_gw1.647.1.1	*Polyporus arcularius*
Polar1_488476	e_gw1.142.26.1	*Polyporus arcularius*
Polar1_143643	CE143642_345	*Polyporus arcularius*
Polar1_524827	estExt_Genewise1.C_2560036	*Polyporus arcularius*
Polar1_492521	e_gw1.231.29.1	*Polyporus arcularius*
Polar1_647821	estExt_fgenesh1_pm.C_4730003	*Polyporus arcularius*
Polar1_645254	estExt_fgenesh1_pm.C_1340008	*Polyporus arcularius*
Polar1_498568	e_gw1.435.13.1	*Polyporus arcularius*
Polar1_648952	estExt_fgenesh1_pm.C_14250001	*Polyporus arcularius*
Polar1_667009	estExt_Genemark1.C_6190005	*Polyporus arcularius*
Trima3_1285902	e_gw1.147.126.1	*Tricholoma matsutake*
Trima3_1260650	e_gw1.61.35.1	*Tricholoma matsutake*
Trima3_1389078	fgenesh1_kg.28_#_152_#_Locus14725v1rp	*Tricholoma matsutake*
Trima3_1384014	fgenesh1_kg.3_#_45_#_Locus14725v1rpkr	*Tricholoma matsutake*
Trima3_1242659	e_gw1.20.174.1	*Tricholoma matsutake*
Trima3_1410082	fgenesh1_kg.273_#_30_#_Locus1429v1rpk	*Tricholoma matsutake*
Trima3_1430874	estExt_Genewise1.C_240383	*Tricholoma matsutake*
Trima3_1295213	e_gw1.187.139.1	*Tricholoma matsutake*
Trima3_1412997	fgenesh1_kg.353_#_1_#_Locus10287v1rpk	*Tricholoma matsutake*
Trima3_1388438	fgenesh1_kg.24_#_73_#_Locus8380v1rpkr	*Tricholoma matsutake*
Pogi_38479513	AAR21094.1	*Pleurotus ostreatus*
Pogi_346305864	AEO22162.1	*Pleurotus ostreatus*
Pogi_40218018	AAR82932.1	*Pleurotus ostreatus*
Pogi_291461620	BAI83290.1	*Pleurotus ostreatus*
Pogi_15594026	CAC69853.1	*Pleurotus ostreatus*
Fvlcc5	AHD24912.1	*Flammulina velutipes*
Fvlcc9	AHD24913.1	*Flammulina velutipes*

Fvlcc1	AAR21096.1	*Flammulina velutipes*
Fvlac1	AHD24914.1	*Flammulina velutipes*
Fvlcc3	AHD24916.1	*Flammulina velutipes*
Fvlcc7	AHD24908.1	*Flammulina velutipes*
Fvlcc8	AHD24909.1	*Flammulina velutipes*
Fvlcc10	AHD24910.1	*Flammulina velutipes*
Fvlcc11	AHD24911.1	*Flammulina velutipes*
Fvlcc4	AHD24917.1	*Flammulina velutipes*
Fvlcc6	AHD24907.1	*Flammulina velutipes*
Fvlac10	KF557733	*Flammulina velutipes*
gfifola_lcc	AHG97584.1	*Grifola frondosa*
pholiota_nameko	ABR24264.1	*Pholiota nameko*
Polacc3	gm1.4288_g	*Pleurotus ostreatus*
Polcc11	e_gw1.06.965.1	*Pleurotus ostreatus*
Polacc12	estExt_fgenesh1_pm.C_110148	*Pleurotus ostreatus*
MCO1_Phanorochaete crsosporium_ferroxidase	AAO42609.1	*Phanorochaete crsosporium*
MCO2_Phanorochaete crsosporium_ferroxidase	AAS21669.1	*Phanorochaete crsosporium*
MCO4_Phanorochaete crsosporium_ferroxidase	AAS21659.1	*Phanorochaete crsosporium*
MCO3_Phanorochaete crsosporium_ferroxidase	AAS21662.1	*Phanorochaete crsosporium*
A_fumigatus_pigment	XP_756088.2	*Aspergillus fumigatus*
A_nidulans_pigment	XP_664239.1	*Aspergillus nidulans*
Aspergillus_Asco	BAA08486.1	*Aspergillus oryzae*
Denbi1_781764	fgenesh1_kg.348_#_1_#_Locus20811v1rpk	*Dendrothele bispora*
Denbi1_964389	estExt_Genemark1.C_830034	*Dendrothele bispora*
Denbi1_817189	fgenesh1_pm.30_#_15	*Dendrothele bispora*
Denbi1_921693	MIX40013_27_80	*Dendrothele bispora*
Denbi1_902236	MIX20556_4_21	*Dendrothele bispora*
Denbi1_821924	fgenesh1_pm.421_#_1	*Dendrothele bispora*
Denbi1_753951	estExt_Genewise1Plus.C_1200016	*Dendrothele bispora*
Denbi1_831303	estExt_fgenesh1_pm.C_5650004	*Dendrothele bispora*
Denbi1_969561	estExt_Genemark1.C_3820012	*Dendrothele bispora*
Denbi1_866476	gm1.19476_g	*Dendrothele bispora*
Phlbr1_25201	fgenesh1_kg.2_#_31_#_isotig05535	*Phlebia brevispora*
Phlbr1_25200	fgenesh1_kg.2_#_30_#_isotig05706	*Phlebia brevispora*
Phlbr1_123463	fgenesh1_pm.2_#_31	*Phlebia brevispora*
Phlbr1_131012	estExt_fgenesh1_pm.C_20032	*Phlebia brevispora*
Phlbr1_76177	e_gw1.9.166.1	*Phlebia brevispora*
Phlbr1_87577	e_gw1.104.3.1	*Phlebia brevispora*
Phlbr1_147965	gm1.5651_g	*Phlebia brevispora*
Phlbr1_126553	fgenesh1_pm.9_#_7	*Phlebia brevispora*
Phlbr1_81224	e_gw1.17.93.1	*Phlebia brevispora*
Phlbr1_27710	fgenesh1_kg.8_#_73_#_isotig04811	*Phlebia brevispora*
U_maydis_mco	XP_761695.1	*Ustilago maydis*
ascorbate oxidase_Acremonium	BAA24288.1	*Acremonium sp*
laccase_Stropharia_aeruginosa	AFE48786.2	*Stropharia_aeruginosa*
laccase_Lentinus_Tigrinus	AY914796.1	*Lentinus_Tigrinus*
Lenti_582048	fgenesh1_kg.104_#_8_#_Locus8932v1rpkr	*Lentinus_Tigrinus*
Ompol1_264	MUStwsD_GLEAN_10008130	*Omphalotus olearius*
Ompol1_2905	MUStwsD_GLEAN_10004978	*Omphalotus olearius*
Ompol1_2807	MUStwsD_GLEAN_10007846	*Omphalotus olearius*
Ompol1_2808	MUStwsD_GLEAN_10007847	*Omphalotus olearius*
Ompol1_4790	MUStwsD_GLEAN_10003836	*Omphalotus olearius*
Ompol1_5400	MUStwsD_GLEAN_10003540	*Omphalotus olearius*
Ompol1_4737	MUStwsD_GLEAN_10000180	*Omphalotus olearius*

Ompol1_5614	MUStwsD_GLEAN_10003324	*Omphalotus olearius*
Monpe1_1_87568	MPER_12490m.01	*Moniliophthora perniciosa*
Monpe1_1_86036	MPER_10857m.01	*Moniliophthora perniciosa*
Monpe1_1_84707	MPER_09368m.01	*Moniliophthora perniciosa*
Monpe1_1_91626	MPER_04426m.01	*Moniliophthora perniciosa*
Monpe1_1_91902	MPER_04733m.01	*Moniliophthora perniciosa*
Monpe1_1_81506	MPER_05846m.01	*Moniliophthora perniciosa*
Monpe1_1_87596	MPER_12520m.01	*Moniliophthora perniciosa*
Monpe1_1_83468	MPER_07993m.01	*Moniliophthora perniciosa*
GYMLU_Lac	KN834794.1	*Gymnopus luxurians*
PLICR_Lac	KN832563.1	*Plicaturopsis crispa*
Meripilus_giganteus_Lac	FR668033.1	*Meripilus_giganteus*
GYMLU_Lac2	KIK67125	*Gymnopus luxurians*
Cerrena_Lac	GQ899201.1	*Cerrena sp.*
GYMLU_Lac3	KIK64093	*Gymnopus luxurians*
Moniliophthora roreri_Lac1	XP_007849995	*Moniliophthora roreri*
Termitomyces_Lac	AB201164.1	*Termitomyces sp.*
Stereum hirsutum_Lac	XM_007311160.1	*Stereum hirsutum*
GYMLU_Lac4	KN834749.1	*Gymnopus luxurians*
Moniliophthora roreri_Lac2	XM_007855266.1	*Moniliophthora roreri*
Termitomyces_Lac2	AB201165.1	*Termitomyces sp.*
GYMLU_Lac5	KN834808.1	*Gymnopus luxurians*
GALMA_Lac6	KL142408.1	*Gymnopus luxurians*
Hebeloma_Lac	KN831778.1	*Hebeloma cylindrosporum*
GYMLU_Lac7	KN834819.1	*Gymnopus luxurians*
Dacryopinax_Lac	JH795872.1	*Dacryopinax sp.*
Gymlu1_173705	e_gw1.46.44.1	*Gymnopus luxurians*
Gymlu1_264864	estExt_Genemark1.C_730040	*Gymnopus luxurians*
Gymlu1_49075	fgenesh1_kg.73_#_30_#_Locus8258v1rpkr	*Gymnopus luxurians*
Gymlu1_77296	fgenesh1_pm.73_#_24	*Gymnopus luxurians*
Gymlu1_155173	e_gw1.5.293.1	*Gymnopus luxurians*
Gymlu1_35304	fgenesh1_kg.3_#_499_#_Locus8460v1rpkr	*Gymnopus luxurians*
Gymlu1_45516	fgenesh1_kg.38_#_183_#_Locus14806v1rp	*Gymnopus luxurians*
Gymlu1_235696	gm1.906_g	*Gymnopus luxurians*
Gymlu1_150929	e_gw1.2.1699.1	*Gymnopus luxurians*
Gymlu1_207072	estExt_Genewise1.C_740128	*Gymnopus luxurians*
Gymlu1_77327	fgenesh1_pm.74_#_24	*Gymnopus luxurians*
Gymlu1_796342	CE530035_1373	*Gymnopus luxurians*
Gymlu1_250550	gm1.15760_g	*Gymnopus luxurians*

Table S3. Summary of purification methods of Lcc2, 4, 5, 13.

	Source	Reference for extraction method	purification steps	N–terminal amino acids
Lcc2	fruiting bodies on day 1 after harvesting	Sakamoto et al. (2005)	Hydrophobic interaction chromatography (Phenyl Sepharose HP column: GE Healthcare, UK) →Anion–exchange chromatography (HiLoad 16/10 Sepharose Q: GE Healthcare, UK) →Gel filtration (Superdex 75 10/30: GE Helthcare, UK) →Anion–exchange chromatography (Mono Q 5/50 GL)	blocked (internal amino acid sequence: YRFRMISIA)
Lcc4	fruiting bodies on day 4 after harvesting	Sakamoto et al. (2005)	Hydrophobic interaction chromatography (Phenyl Sepharose HP column: GE Healthcare, UK) →Anion–exchange chromatography (HiLoad 16/10 Q: GE Healthcare, UK) →Gel filtration (Superdex 75 10/30: GE Helthcare, UK)	blocked (internal amino acid sequence: LDIIRNNGVLQIVNR)
Lcc5	sawdust media under colony edge	Nagai et al. (2009)	anion–exchange chromatography (HiLoad 16/10 Sepharose Q: GE Healthcare, UK) →hydrophobic interaction chromatography (HiLoad 16/10 Phenyl Sepharose: GE Healthcare, UK)	VLETTGDLVISNAAV
Lcc13	mycelia on sawdust media described in Material and methods	Nagai et al. (2009)	Hydrophobic interaction chromatography (HiLoad 16/10 Phenyl Sepharose: GE Healthcare, UK) →Anion–exchange chromatography (HiLoad 16/10 Sepharose Q: GE Healthcare, UK)	XTLPITLNIVNAQGA

Table S4 Primers used for mapping

name	forward (5'–3')	reverse (5'–3')	method
lcc1	GACGGGTATGCAAATACTTTTCCC	CATACCCCAGCTTGGTCCGGAAC	SSCP
lcc2	CACCAGGACCGAGCTTATCGC	CGCTGGCCAGGGAAGATCTG	SSCP
lcc3	AATAAGGACATTGCTCCAGACGG	CGTTGATCGTGAATCTATCGCCC	SSCP
lcc4	TGTGTTTCACCGCTGTCACTCC	TTTCTCGGCACACAATCATCG	SSCP
lcc5	GACGGACATAAACACATAGAACG	ACAACTGACTCTCATGCAAGCG	ASP
lcc6	AGCCGAAAATCGTGCGTCC	ATCCATGCCAGTGCTAAGCG	ASP
lcc7	CGAAACGGGCTGGTCTGACC	GGTTGGGTGTTAACTCCGTC	SSCP
lcc9	GTAAGCCCAGTGCTCCTGCG	ACATTTTGCCAGATCGGCCG	SSCP
Lcc10	CCATTTGGTTTCAGATATAC	AGTATGCTCTTTAGCTAAAG	ASP
Lcc11	ATATCCTATCCTATCCTATC	TCCATTTATTTGCCCTCCCC	ASP
lcc12	GTAGCGTCTGTCTATAGCGAC	GCCTATCCTGGATTGGTCGG	SSCP
lcc13	TGTGTGCCTGCAGTACCACG	CACGTGCCAGCTTTCGAGG	SSCP
lcc14	AAGTCTTGGCCTGCAGGAGC	TTCTTGGGACTGAACCTGCC	SSCP
tyr	AAGCGATGCCTCTTCGCACCTGGTAGACAG	AAAATGGGGATCAGTAGCCCATTCC	SSCP

SSCP: Single Strand Conformation Polymorphic

ASP: Allele Specific PCR

Table S5 Primers used in real-time PCR

primer name	sequence
Lcc1-rtU	ACGTCGCCGCCGTTAAT
Lcc1-rtL	GCATCATAAGTTGGGCAAAGTTG
Lcc2-rtU	AAGCTCCCTCTCTAGTCAAAGCAA
Lcc2-rtL	GCTGTCGTAAATGGGACAAAGTT
Lcc3-rtU	CCCTGCTCAACGGTACTCATTC
Lcc3-rtL	CTCGGATGCGGTAGTTGGA
Lcc4-rtU	GCTGGCCTCGCAGTTGTT
Lcc4-rtL	CAACGGAACTGGATTGTTGGA
Lcc5-rtU	CAGCGGTGCGGAAACG
Lcc5-rtL	TGGTGGGCAAGGAGTAAACAG
Lcc6-rtU	CATCTGGAGGCTGGATTTGC
Lcc6-rtL	TTGTCGGATTAGCGGAAGCA
Lcc7-rtU	CGTTCTCGCTGGATTATCTTCTC
Lcc7-rtL	CGGTCCCCTTTGGTTCCT
Lcc9-rtU	GTAGAAGTATGGTGACCGCTAATGG
Lcc9-rtL	GCCCTTGCTCGCAGTGAT
Lcc10_rtU	AGCGCAACAGTATGCCGATA
Lcc10_rtL	CGAGGGCTTCGCAATGG
Lcc11_rtU	CTCCTGCGGACGAGACCTT
Lcc11_rtL	CCCCGTCGCAGTTCGA
Lcc12-rtU	AACGCCACTATCGAAGTCTCTATTC
Lcc12-rtL	TGTGACCGTGCAAGTGGAA
Lcc13-rtU	CCAGGAACTTGAACCAAAACG
Lcc13-rtL	GGCGCACCTTTGTATCGTAAG
Lcc14-rtU	CCTCCGCGAGAAACGTATTTA
Lcc14-rtL	GTCGGTTTCGATGATAGTCATGTT

Table S6 Summary of previous research on laccases of *Lentinula edodes*.

	gene (publication)	Accession number (DDBJ)	enzyme
Lcc1	Sakamoto et al. 2008	AB035409 (Sato 1999)	Nagai et al. 2002, Wong et al. 2013
Lcc2	Wong et al. 2013	AB035410 (Sato and Hirano 1999)	this paper (different enzyme purified by Nagai et al. 2003, designated Lcc2)
Lcc3	Wong et al. 2013	AB046713 (Sato and Irie 2001)	not purified
Lcc4	Sakamoto et al. 2009,	AB446445 (Sakamoto et al. 2008)	Nagai et al. 2003 (designated as Lcc2), Yano et al. 2009, Wong et al. 2013
Lcc5	Wong et al. 2013	AB543788 (Sakamoto et al. 2010)	Wong et al. 2013, This paper
Lcc6	this paper	AB543787 (Sakamoto et al. 2010)	Nagai et al. 2009
Lcc7	Wong et al. 2013	JX879731 (Wong et al. 2013)	Wong et al. 2013
Lcc8	Wong et al. 2013	JX879732 (Wong et al. 2013)	not purified
Lcc9	Wong et al. 2013	JX879733 (Wong et al. 2013)	not purified
Lcc10	Wong et al. 2013	JX879734 (Wong et al. 2013)	not purified
Lcc11	Wong et al. 2013	JX879735 (Wong et al. 2013)	not purified
Lcc12	this paper	this paper	not purified
Lcc13	this paper	this paper	this paper
Lcc14	this paper	this paper	not purified

Table S7 Number of cis-elements in laccase promoters and number of introns in laccase genes in *L. edodes*.

	Metal response element (MRE) TGCRCNC	Xenobiotic response element (XRE) CACGC	activator of Cup element (ACE1) NTNNHGCTG	CAAT box CAAT	CreA-binding site (CreA) SYGGRG	numbers of introns
lcc1	0	1	1(c:1)	15	7(c:3)	27
lcc2	1	1(c:1)	5(c:2)	27	3(c:2)	12
lcc3	3(c:1)	0	2(c:1)	12	2(c:1)	11
lcc4	1(c:1)	1(c:1)	2(c:2)	10	2	16
lcc5	2	1	2	13	2	20
lcc6	0	2(c:1)	4(c:3)	7	1	27
lcc7	3(c:3)	5(c:1)	1(c:1)	4	6(c:3)	11
lcc9	1(c:1)	5(c:3)	3(c:1)	11	6(c:4)	14
lcc10	1	3(c:1)	4(c:2)	11	5(c:4)	9
lcc11	0	2(c:1)	5(c:3)	15	3(c:1)	8
lcc12	1	1	3(c:1)	11	4(c:2)	17
lcc13	0	1	0	1	7(c:5)	13
lcc14	1(c:1)	1(c:1)	3(c:2)	9	0	9

c: number of complementary strand sequences
